# *BRCA1* testing should be offered to individuals with triple-negative breast cancer diagnosed below 50 years

**DOI:** 10.1038/bjc.2012.31

**Published:** 2012-02-14

**Authors:** L Robertson, H Hanson, S Seal, M Warren-Perry, D Hughes, I Howell, C Turnbull, R Houlston, S Shanley, S Butler, D G Evans, G Ross, D Eccles, A Tutt, N Rahman, TNT Trial TMG, BCSC (UK)

**Affiliations:** 1 Division of Genetics and Epidemiology, Institute of Cancer Research, 15 Cotswold Road, Sutton, Surrey SM2 5NG, UK; 2 Department of Cancer Genetics, The Royal Marsden NHS Foundation Trust, Downs Road, Sutton SM2 5PT, UK; 3 SW Thames Regional Genetics Service, St George's University of London, St George's Hospital, Tooting, London SW17 0RE, UK; 4 Department of Genetic Medicine, St Mary's Hospital, Manchester Academic Health Science Centre, Manchester M13 9WL, UK; 5 Breast Cancer Unit, Royal Marsden NHS Foundation Trust, London SW3 6JJ, UK; 6 Human Cancer Sciences Division, Faculty of Medicine, University of Southampton, Southampton University Hospitals NHS Trust, Southampton SO16 6YD, UK; 7 Breakthrough Breast Cancer Research Unit, King's College, London SE1 9RT, UK

**Keywords:** triple-negative, BRCA1, breast cancer, genetic testing

## Abstract

**Background:**

Triple-negative (TN) tumours are the predominant breast cancer subtype in *BRCA1* mutation carriers. Recently, it was proposed that all individuals below 50 years of age with TN breast cancer should be offered BRCA testing. We have evaluated the *BRCA1* mutation frequency and the implications for clinical practice of undertaking genetic testing in women with TN breast cancer.

**Methods:**

We undertook *BRCA1* mutation analysis in 308 individuals with TN breast cancer, 159 individuals from unselected series of breast cancer and 149 individuals from series ascertained on the basis of young age and/or family history.

**Results:**

*BRCA1* mutations were present in 45 out of 308 individuals. Individuals with TN cancer <50 years had >10% likelihood of carrying a *BRCA1* mutation in both the unselected (11 out of 58, 19%) and selected (26 out of 111, 23%) series. However, over a third would not have been offered testing using existing criteria. We estimate that testing all individuals with TN breast cancer <50 years would generate an extra 1200 tests annually in England.

**Conclusion:**

Women with TN breast cancer diagnosed below 50 years have >10% likelihood of carrying a *BRCA1* mutation and are therefore eligible for testing in most centres. However, implementation may place short-term logistical and financial burdens on genetic services.

Triple-negative (TN) breast cancer describes a subgroup of tumours that lack expression of oestrogen receptor (ER), progesterone receptor (PR) and human epidermal growth factor receptor (HER2) (Foulkes *et al*, 2010). Overall, TN cancers account for about 15% of all breast cancers, but occur more frequently in younger women and are the predominant cancer subtype in individuals with a germline *BRCA1* mutation (Bauer *et al*, 2007; Atchley *et al*, 2008; Blows *et al*, 2010; Foulkes *et al*, 2010).

The identification of a BRCA mutation has profound consequences for clinical management; impacting on the likelihood of developing contralateral breast cancer and/or ovarian cancer and increasingly having implications for optimal therapy (Antoniou *et al*, 2003; Fong *et al*, 2009; Tutt *et al*, 2010; Nathanson and Domchek, 2011). Due to financial and logistical constraints, BRCA testing is currently rationed in most countries. In the US and much of Europe, BRCA testing is typically undertaken if the likelihood of detecting a mutation is >10% (American Society of Clinical Oncology, 2003; Gadzicki *et al*, 2011). In the UK, the National Institute for Health and Clinical Excellence (NICE) recommended that testing should minimally be offered if the likelihood of detecting a mutation is >20%, though many UK centres also offer testing if the likelihood is between 10–20%, (McIntosh *et al*, 2004; NICE, 2006). Several different methods to determine which cases are eligible for testing are utilised in clinical practice, most of which require specialised knowledge and/or software (Antoniou *et al*, 2008).

The recognition of the strong association of the TN phenotype and *BRCA1* mutations has led to efforts for establishing the frequency of *BRCA1* mutations in individuals with TN breast cancer. To date, several small studies have evaluated this in both unselected series and case series selected on the basis of family history and/or age (Table 1). Additionally, it was recently proposed that BRCA testing all women with TN cancers diagnosed below 50 years would be cost-effective with respect to overall health spending at a national level (Kwon *et al*, 2010), based on an estimated mutation prevalence of 10–25%. However, this study did not address the practical and cost implications for the local services that undertake testing.

In this study, we have undertaken *BRCA1* analysis in 308 individuals with TN breast cancer; the largest study to date. We have used the data to further evaluate the mutation frequency and to consider the practical ramifications of undertaking BRCA testing in individuals with TN breast cancer.

## Materials and methods

### Cases

We included 308 TN breast cancers from UK. Oestrogen receptor, PR and HER2 status were confirmed either in a histopathology report and/or a clinician's referral letter. When not explicitly stated, ER and PR status were scored as negative when there was absent expression (equivalent to a Quickscore of 0 out of 8). Human epidermal growth factor receptor was regarded as negative when scored as 0 or 1+ for HER2 by immunohistochemistry and/or when there was non-amplification of HER2 by fluorescent *in situ* hybridisation.

The cases were either from case series unselected with respect to genetic susceptibility (the unselected series *n*=159) or from case series that were specifically ascertained because of young age at diagnosis and/or a family history of breast cancer (the selected series *n*=149). The unselected series came from either the ongoing TNT trial ISRCTN97330959 (Kilburn, 2008), a UK-wide randomised phase III trial of carboplatin compared with docetaxel for patients with metastatic or recurrent locally advanced TN breast cancer (*n*=81); or the Marsden sample series, which was a collection of samples from breast cancer patients attending the oncology clinics at the Marsden Hospital (*n*=78). The selected series came from either the Familial Breast Cancer Study (*n*=90), from referrals to our regional genetics department (*n*=25) or from the Prospective study of Outcomes in Sporadic versus Hereditary breast cancer (POSH, *n*=34). The latter was a UK-wide study that recruited individuals with invasive breast cancer aged ⩽40 years (Eccles *et al*, 2007). None of the cases have been included in any other published study on TN breast cancer. The study was undertaken as part of our research into the genetic causes of breast cancer, which has been approved by the London Multicentre Research Ethics Committee (MREC/01/2/18).

### *BRCA1* analysis

*BRCA1* mutation analysis, including multiplex ligation-dependent probe amplification (MLPA) analysis for large deletions/duplications, was performed in DNA from all cases. This was either performed through a clinical *BRCA* test by the local centre, or was undertaken by ourselves by sequencing genomic DNA through the 24 coding exons and intron–exon boundaries of *BRCA1* and undertaking MLPA using probe mix P002. All mutations were confirmed by separate bi-directional sequencing in a second sample. All copy number changes were confirmed in a fresh aliquot of DNA with a different probe mix (P087). The mutation nomenclature is in accordance with HGVS convention with numbering starting at the first A of the ATG initiation site, using U14680.1 as the reference sequence.

### Assessment of eligibility for clinical BRCA testing

Currently, in UK there is variability in the threshold used for BRCA testing; in the South West Thames Regional Genetics and Royal Marsden Cancer Genetics services we use a threshold of 10% and primarily use the Manchester score to assess this (Evans *et al*, 2005). Family history information was available for 271 individuals. We calculated the Manchester score, and designated those with a score ⩾15, as eligible for clinical testing (Supplementary Tables 1 and 2). Of note, this classification does not necessarily mean that these cases actually had clinical BRCA testing; for many, this information was not known and in some cases we were aware that testing had not occurred. The classification denotes whether the patient would have been eligible for testing by genetics departments operating a 10% mutation detection threshold.

### Age-specific TN breast cancer incidence

National breast cancer figures in England are not subclassified by receptor status. Therefore, to estimate the annual age-specific incidence of TN breast cancer in England we used the national figures for age-specific breast cancer incidence published by the Office for National Statistics for England (Office for National Statistics, 2006) and data from a study of 10 159 cases of breast cancer subtyped by immunohistochemistry (Blows *et al*, 2010). This allowed us to estimate the annual age-specific incidence of TN breast cancer in England.

## Results

### *BRCA1* mutation frequency

The full results of all 308 cases are given in Supplementary Table 1. Overall, there were 45 *BRCA1* mutations in the 308 individuals (14.6%). This included 15 (9.4%) *BRCA1* mutations in 159 individuals in the unselected series, and 30 (20.1%) *BRCA1* mutations in 149 individuals in the selected series (Table 2). There was a strong age-effect with marked decrease in mutation frequency in individuals aged over 50 years in both the unselected and selected series (Table 2). If one considers just individuals with sporadic TN breast cancer, that is, those without a first or second degree relative with breast or ovarian cancer, 8 out of 103 (8%) had a *BRCA1* mutation, and all were under 50 years of age.

### Eligibility for clinical BRCA testing

Family history data was available in 271 individuals, which included 122 out of 159 individuals in the unselected series and all individuals in the selected series. We used these data to calculate the Manchester score for each family to evaluate whether the individual would be eligible for clinical BRCA testing (Manchester score ⩾15, Supplementary Table 2). Overall, approximately a third of cases (85 out of 271; 31%) were eligible for clinical BRCA gene testing, though as expected this was lower in the unselected series (20 out of 122; 16%), particularly at older ages (Supplementary Table 1). If one considers the individuals with *BRCA1* mutations for whom we could calculate the Manchester score, 15 out of 42 (36%) were not eligible for clinical BRCA testing (Table 3).

### Impact of using age-specific testing threshold on BRCA testing

To estimate the number of BRCA tests that would be undertaken in TN cases, if age-specific criteria were employed, we used the cancer registration statistics from the Office for National Statistics for England (Office for National Statistics, 2006), together with the estimates of the proportion of breast cancers that are TN by age (Blows *et al*, 2010). These suggest that ∼6000 women with TN breast cancer are diagnosed each year in England, of which ∼500 are <40 years of age and ∼1500 are <50 years of age (Table 4). If one assumes that the data from the unselected series are broadly representative of the proportion of these cases that would not currently be eligible for clinical BRCA testing, 69% (11 out of 16) of TN <40 years, 77% (33 out of 43) <50 years, 79% (72 out of 91) <60 years and (84%) (102 out of 122) of all TN cases would not be eligible for clinical BRCA testing (percentages reflect the number of individuals not eligible for testing/total number of individuals in that age group for whom eligibility status was known, see Supplementary Table 1). If all individuals with TN breast cancer diagnosed before 50 years of age were tested, these data suggest that an additional ∼1200 BRCA tests would be performed per year in England.

## Discussion

We have undertaken the largest analysis of *BRCA1* in TN breast cancer to date, and showed that the frequency of *BRCA1* mutations in unselected individuals with TN breast cancer is ∼10%, increasing to ∼19% of individuals diagnosed below 50 years. The latter is similar to the frequency of *BRCA1* mutations (23%) in individuals diagnosed before 50 years that were selected for inclusion because of a family history of breast cancer and/or young age at diagnosis. Our study included samples from four different sources and more precise figures would be obtainable from larger, prospective studies. Nevertheless, our results are similar to those previously reported and we believe that they are likely to be broadly accurate (refs in Table 1).

We estimated the proportion of individuals included in this study that would currently qualify for a clinical BRCA test. Our analysis suggests that over a third of the *BRCA1* mutation-positive individuals we identified would not have been eligible for clinical genetic testing in departments that use a 10% mutation detection threshold calculated without consideration of histological parameters.

Taken together, these data strongly indicate that histological parameters should be included in deciding which individuals should be offered BRCA testing. There have been efforts to incorporate histological parameters into existing BRCA-testing selection systems, such as BOADICEA and the Manchester system, (Evans *et al*, 2009; Mavaddat *et al*, 2010). Additionally, studies to define testing criteria in individuals that are not eligible for testing using current systems (e.g., individuals with TN breast cancer but without a family history) have been undertaken (Young *et al*, 2009; Evans *et al*, 2011). We believe a drawback to these approaches is that complex evaluation by specialist practitioners is typically required for most cases, but is unnecessary in the sizeable proportion eligible for testing on the basis of age alone.

A simpler approach, which would be readily comprehensible by clinicians and patients, would be to define a BRCA testing eligibility criteria for women with TN breast cancer based on age. Only individuals above the age threshold would require the more detailed, specialist review incorporating family history and scoring algorithms to decide whether they were eligible for BRCA testing.

Our data suggests that diagnosis of TN cancer below 50 years would be a suitable age threshold for BRCA testing. It is also consistent with recent simulation data, suggesting that this testing threshold would be a cost-effective strategy and would result in substantial reduction in subsequent breast and ovarian cancer in mutation-positive women (Kwon *et al*, 2010). However, implementation of routine BRCA testing in all TN breast cancers diagnosed before 50 years would increase the logistical and financial burdens on genetic departments. We estimate that it would lead to ∼1200 extra tests in England each year, which may be challenging for some departments to immediately implement with current resources and procedures. However, new sequencing technologies are leading us into an era of fast, affordable gene testing. Together with procedural reorganisation to allow BRCA testing in affected individuals to be undertaken though oncology services (with support from genetics as required), this should enable genetic services to introduce BRCA testing to women with TN breast cancer diagnosed below 50 years of age, within the next few years.

## Figures and Tables

**Table 1:** Studies with over 50 cases that have evaluated *BRCA1* mutation prevalence in TN cancers

**Number of cases**	**BRCA1 mutations (%)**	**Unselected/selected**	**Selection criteria**	**Reference**
144	20 (14)	Unselected		Collins *et al* (2009)
96	9 (9)	Selected	Bilateral and/or family history of breast cancer.	Zhang *et al* (2011)
93	32 (34)	Selected	Seen in Genetic clinics and underwent BRCA testing.	Atchley *et al* (2008)
77	12 (16)	Unselected		Gonzalez-Angulo *et al* (2011)
64	19 (30)	Selected	Ashkenazi Jewish heritage. Tested for founder mutations.	Comen *et al* (2011)
63	8 (13)	Selected and unselected	TN <41 years	Evans *et al* (2011)
54	5 (9)	Selected	TN <40 years *and* did not qualify for testing according to ASCO guidelines	Young *et al* (2009)

Abbreviation: TN=Triple-negative.

**Table 2:** Summary of *BRCA1* mutations in 308 TN breast cancer cases

	**Unselected series *BRCA1* mut/all (%)**	**Selected series *BRCA1* mut/all (%)**	**Total *BRCA1* mut/all (%)**
All	15/159 (9)	30/149 (20)	45/308 (15)
<50 years	11/58 (19)	26/111 (23)	37/169 (22)
⩾50 years	4/101 (4)	4/38 (11)	8/139 (6)

Abbreviation: TN=Triple-negative.

**Table 3:** Eligibility for clinical BRCA testing by age and mutation status

	**Unselected series MS ⩾15/all (%)**	**Selected series MS ⩾15/all (%)**	**Total MS ⩾15/all (%)**
*All*	20/122 (16)	65/149 (43)	85/271 (31)
<40 Years	5/16 (31)	28/78 (39)	33/94 (35)
<50 Years	10/43 (23)	45/111 (40)	55/154 (36)
			
*All BRCA1 mutations*	7/12 (58)	20/30 (67)	27/42 (64)
BRCA1 mut <40 years	2/3 (67)	8/15 (53)	10/18 (55)
BRCA1 mut <50 years	5/8 (63)	17/26 (65)	22/34 (65)

Abbreviation: MS=Manchester score.

**Table 4:** Age-specific incidence of TN breast cancer and impact on BRCA testing

**Age**	**Total cases in England^a^**	**Proportion that are TN^b^ (%)**	**TN cases per year**	**Proportion not eligible for BRCA testing (%)**	**Additional BRCA tests per year, if all TN tested**
<40 Years	1765	29	512	69	353
<50 Years	7384	21	1551	77	1194
<60 Years	16156	17	2747	79	2170
All	38004	16	6081	84	5108

Abbreviation: TN=Triple-negative.

a Office for National Statistics (2006).

b Extrapolated from the data in Blows *et al* (2010).
