# Supplementary material for: BRCA1 testing should be offered to individuals with triple-negative breast cancer diagnosed below 50 years
Source: Br J Cancer. 2012 Feb 14;106(6):1234–8. doi: 10.1038/bjc.2012.31 (PMC3304410; doi:10.1038/bjc.2012.31)
Supplement: Supplementary Information [file bjc201231x1.doc]

**Supplementary Information:**

***BRCA1* testing should be offered to individuals with triple-negative breast cancer diagnosed below 50 years**

Lisa Robertson^1,2^, Helen Hanson^1,3^, Sheila Seal^1^, Margaret Warren-Perry^1^, Deborah Hughes^1^, Imran Howell^1^, Clare Turnbull^1^, Richard Houlston^1^, Sue Shanley^2^, Samantha Butler^3^, BCSC (UK), TNT Trial TMG, D Gareth Evans^4^, Gill Ross^5^, Diana Eccles^6^, Andrew Tutt^7^ and Nazneen Rahman^1,2^

**Supplementary Table 1: Characteristics and *BRCA1*** mutation analysis of 308 triple-negative breast cancer cases

| **ID** | **Age at Diagnosis** | **Selected / Unselected** | **Eligible for clinical BRCA test** | **BRCA1 mutation** |
| --- | --- | --- | --- | --- |
| 1 | 22 | selected | no | c.2269delG |
| 2 | 23 | selected | no |  |
| 3 | 25 | selected | yes |  |
| 4 | 25 | selected | no |  |
| 5 | 25 | selected | no |  |
| 6 | 25 | selected | no |  |
| 7 | 26 | selected | no | c.2679_2682delGAAA |
| 8 | 27 | selected | yes | c.68_69delAG |
| 9 | 27 | selected | no | c.3756_3759delGTCT |
| 10 | 27 | selected | yes | c.2955delC |
| 11 | 27 | selected | no |  |
| 12 | 27 | selected | no |  |
| 13 | 27 | selected | yes |  |
| 14 | 27 | selected | no |  |
| 15 | 27 | selected | yes |  |
| 16 | 28 | selected | yes | c.3331_3334delCAAG |
| 17 | 28 | selected | no | c.2612delCinsTT |
| 18 | 28 | selected | no |  |
| 19 | 28 | selected | no |  |
| 20 | 28 | selected | no |  |
| 21 | 29 | selected | no | c.4185+1G>T |
| 22 | 29 | selected | no |  |
| 23 | 29 | selected | no |  |
| 24 | 29 | selected | no |  |
| 25 | 29 | selected | no |  |
| 26 | 29 | selected | no |  |
| 27 | 30 | selected | no | exon 17-19 deletion |
| 28 | 30 | selected | no | c.4065_4068delTCAA |
| 29 | 30 | selected | no |  |
| 30 | 30 | selected | yes |  |
| 31 | 30 | selected | no |  |
| 32 | 30 | selected | no |  |
| 33 | 30 | selected | no |  |
| 34 | 30 | selected | no |  |
| 35 | 30 | selected | no |  |
| 36 | 30 | selected | no |  |
| 37 | 30 | selected | yes |  |
| 38 | 30 | selected | no |  |
| 39 | 30 | selected | no |  |
| 40 | 30 | selected | yes |  |
| 41 | 31 | selected | yes | exon 17 deletion |
| 42 | 31 | selected | yes | c.5068A>T_p.Lys1690X |
| 43 | 31 | selected | yes |  |
| 44 | 31 | selected | yes |  |
| 45 | 31 | unselected | no |  |
| 46 | 31 | unselected | no |  |
| 47 | 32 | selected | yes |  |
| 48 | 32 | unselected | no |  |
| 49 | 32 | selected | no |  |
| 50 | 32 | selected | no |  |
| 51 | 32 | selected | yes |  |
| 52 | 32 | selected | yes |  |
| 53 | 33 | selected | no |  |
| 54 | 33 | unselected | unknown |  |
| 55 | 33 | selected | yes |  |
| 56 | 33 | selected | no |  |
| 57 | 34 | unselected | unknown | c.5194-2A>C |
| 58 | 34 | selected | yes |  |
| 59 | 34 | unselected | no |  |
| 60 | 34 | selected | no |  |
| 61 | 34 | unselected | unknown |  |
| 62 | 34 | selected | no |  |
| 63 | 35 | selected | yes | c.4760C>G_p.Ser1587X |
| 64 | 35 | unselected | no |  |
| 65 | 35 | selected | no |  |
| 66 | 35 | selected | yes |  |
| 67 | 35 | selected | yes |  |
| 68 | 35 | selected | no |  |
| 69 | 35 | selected | no |  |
| 70 | 35 | selected | no |  |
| 71 | 35 | selected | yes |  |
| 72 | 35 | selected | no |  |
| 73 | 35 | unselected | yes |  |
| 74 | 36 | selected | no |  |
| 75 | 36 | selected | yes |  |
| 76 | 36 | unselected | unknown |  |
| 77 | 36 | unselected | yes |  |
| 78 | 37 | selected | yes | c.2011insT |
| 79 | 37 | unselected | no |  |
| 80 | 37 | unselected | no |  |
| 81 | 37 | selected | no |  |
| 82 | 37 | selected | yes |  |
| 83 | 37 | unselected | no |  |
| 84 | 37 | selected | no |  |
| 85 | 37 | selected | no |  |
| 86 | 38 | unselected | no | c.4065_4068delTCAA |
| 87 | 38 | unselected | yes | c.1687C>T_p.Gln563X |
| 88 | 38 | selected | yes |  |
| 89 | 38 | selected | no |  |
| 90 | 38 | selected | yes |  |
| 91 | 39 | selected | yes | exon 3 deletion |
| 92 | 39 | unselected | yes | c.639delC |
| 93 | 39 | selected | no |  |
| 94 | 39 | selected | no |  |
| 95 | 39 | selected | no |  |
| 96 | 39 | unselected | yes |  |
| 97 | 39 | unselected | no |  |
| 98 | 39 | unselected | no |  |
| 99 | 40 | selected | yes | exon 13 duplication |
| 100 | 40 | unselected | unknown | c.3331_3334delCAAG |
| 101 | 40 | selected | yes |  |
| 102 | 40 | selected | no |  |
| 103 | 40 | unselected | no |  |
| 104 | 41 | selected | yes | c.5193+2delT |
| 105 | 41 | unselected | no |  |
| 106 | 41 | unselected | no |  |
| 107 | 41 | unselected | no |  |
| 108 | 41 | selected | no |  |
| 109 | 41 | unselected | no |  |
| 110 | 41 | unselected | no |  |
| 111 | 42 | selected | no | exon 22 deletion |
| 112 | 42 | selected | yes | c.3416delG |
| 113 | 42 | selected | yes | c.1175_1214del40 |
| 114 | 42 | selected | yes |  |
| 115 | 42 | selected | no |  |
| 116 | 42 | unselected | no |  |
| 117 | 42 | selected | no |  |
| 118 | 43 | selected | yes |  |
| 119 | 43 | unselected | unknown |  |
| 120 | 43 | selected | no |  |
| 121 | 43 | unselected | unknown |  |
| 122 | 43 | unselected | no |  |
| 123 | 44 | unselected | yes | c.68_69delAG |
| 124 | 44 | unselected | no | c.2612delCinsTT |
| 125 | 44 | selected | no | c.2253_2254delGT |
| 126 | 44 | unselected | unknown |  |
| 127 | 44 | unselected | no |  |
| 128 | 44 | selected | no |  |
| 129 | 44 | unselected | no |  |
| 130 | 44 | unselected | no |  |
| 131 | 45 | selected | yes | c.68_69delAG |
| 132 | 45 | unselected | yes | c.2766delA |
| 133 | 45 | unselected | unknown |  |
| 134 | 45 | unselected | no |  |
| 135 | 45 | unselected | unknown |  |
| 136 | 45 | selected | no |  |
| 137 | 45 | unselected | no |  |
| 138 | 46 | selected | yes | exon 13 duplication |
| 139 | 46 | selected | yes | exon 1-23 deletion |
| 140 | 46 | unselected | yes | c.4574_4575delAA |
| 141 | 46 | unselected | unknown | c.2679_2682delGAAA |
| 142 | 46 | unselected | unknown |  |
| 143 | 46 | selected | no |  |
| 144 | 46 | selected | yes |  |
| 145 | 46 | unselected | no |  |
| 146 | 46 | unselected | no |  |
| 147 | 46 | unselected | no |  |
| 148 | 47 | selected | yes | c.3770_3771delAG |
| 149 | 47 | unselected | no |  |
| 150 | 47 | unselected | no |  |
| 151 | 47 | selected | no |  |
| 152 | 47 | unselected | no |  |
| 153 | 47 | selected | no |  |
| 154 | 47 | selected | no |  |
| 155 | 47 | unselected | no |  |
| 156 | 48 | selected | yes | c.5177_5180delGAAA |
| 157 | 48 | selected | yes |  |
| 158 | 48 | selected | no |  |
| 159 | 48 | selected | yes |  |
| 160 | 48 | unselected | unknown |  |
| 161 | 49 | unselected | no | exon 13 duplication |
| 162 | 49 | unselected | yes |  |
| 163 | 49 | selected | yes |  |
| 164 | 49 | unselected | unknown |  |
| 165 | 49 | unselected | unknown |  |
| 166 | 49 | selected | no |  |
| 167 | 49 | selected | no |  |
| 168 | 49 | selected | yes |  |
| 169 | 49 | unselected | yes |  |
| 170 | 50 | unselected | yes | exon 20 deletion |
| 171 | 50 | selected | no |  |
| 172 | 50 | selected | yes |  |
| 173 | 50 | selected | no |  |
| 174 | 50 | selected | no |  |
| 175 | 50 | unselected | no |  |
| 176 | 50 | selected | no |  |
| 177 | 51 | unselected | no |  |
| 178 | 51 | unselected | no |  |
| 179 | 51 | unselected | no |  |
| 180 | 51 | unselected | no |  |
| 181 | 51 | unselected | unknown |  |
| 182 | 51 | unselected | no |  |
| 183 | 52 | selected | yes | c.2681_ 2682delAA |
| 184 | 52 | selected | yes |  |
| 185 | 52 | unselected | no |  |
| 186 | 52 | selected | yes |  |
| 187 | 52 | unselected | no |  |
| 188 | 52 | unselected | unknown |  |
| 189 | 52 | unselected | no |  |
| 190 | 53 | unselected | no |  |
| 191 | 53 | selected | yes |  |
| 192 | 53 | selected | no |  |
| 193 | 53 | unselected | unknown |  |
| 194 | 53 | selected | yes |  |
| 195 | 53 | unselected | no |  |
| 196 | 53 | selected | no |  |
| 197 | 53 | unselected | no |  |
| 198 | 53 | unselected | no |  |
| 199 | 54 | selected | yes |  |
| 200 | 54 | unselected | unknown |  |
| 201 | 54 | unselected | unknown |  |
| 202 | 54 | selected | yes |  |
| 203 | 54 | unselected | yes |  |
| 204 | 54 | unselected | no |  |
| 205 | 54 | unselected | no |  |
| 206 | 54 | unselected | no |  |
| 207 | 55 | unselected | no | exon 13 duplication |
| 208 | 55 | unselected | no | c.2561dupGC |
| 209 | 55 | unselected | no |  |
| 210 | 55 | unselected | no |  |
| 211 | 55 | selected | yes |  |
| 212 | 55 | selected | yes |  |
| 213 | 55 | unselected | no |  |
| 214 | 55 | unselected | no |  |
| 215 | 55 | selected | yes |  |
| 216 | 55 | unselected | no |  |
| 217 | 55 | unselected | unknown |  |
| 218 | 55 | unselected | unknown |  |
| 219 | 55 | unselected | no |  |
| 220 | 56 | selected | yes | c.547+1G>T |
| 221 | 56 | unselected | no |  |
| 222 | 56 | unselected | yes |  |
| 223 | 56 | unselected | no |  |
| 224 | 56 | unselected | unknown |  |
| 225 | 56 | unselected | no |  |
| 226 | 57 | unselected | no |  |
| 227 | 57 | unselected | unknown |  |
| 228 | 57 | unselected | unknown |  |
| 229 | 57 | unselected | yes |  |
| 230 | 57 | unselected | no |  |
| 231 | 57 | selected | no |  |
| 232 | 57 | unselected | yes |  |
| 233 | 58 | unselected | yes |  |
| 234 | 58 | unselected | no |  |
| 235 | 58 | unselected | unknown |  |
| 236 | 58 | unselected | no |  |
| 237 | 58 | unselected | no |  |
| 238 | 58 | unselected | no |  |
| 239 | 58 | unselected | no |  |
| 240 | 58 | unselected | no |  |
| 241 | 58 | unselected | no |  |
| 242 | 59 | unselected | yes | c.3756_3759delGTCT |
| 243 | 59 | selected | yes |  |
| 244 | 59 | unselected | yes |  |
| 245 | 59 | unselected | no |  |
| 246 | 59 | selected | no |  |
| 247 | 59 | unselected | unknown |  |
| 248 | 59 | selected | no |  |
| 249 | 59 | unselected | yes |  |
| 250 | 59 | unselected | no |  |
| 251 | 59 | unselected | no |  |
| 252 | 60 | selected | yes |  |
| 253 | 60 | unselected | no |  |
| 254 | 61 | selected | no | c.302-2A>G |
| 255 | 61 | unselected | unknown |  |
| 256 | 61 | unselected | no |  |
| 257 | 61 | unselected | unknown |  |
| 258 | 61 | unselected | no |  |
| 259 | 62 | selected | no |  |
| 260 | 62 | unselected | no |  |
| 261 | 63 | unselected | no |  |
| 262 | 63 | selected | yes |  |
| 263 | 63 | unselected | unknown |  |
| 264 | 64 | selected | yes | c.302-3C>G |
| 265 | 64 | selected | no |  |
| 266 | 64 | unselected | no |  |
| 267 | 64 | unselected | yes |  |
| 268 | 64 | unselected | unknown |  |
| 269 | 65 | unselected | no |  |
| 270 | 65 | unselected | unknown |  |
| 271 | 65 | unselected | no |  |
| 272 | 65 | unselected | no |  |
| 273 | 66 | unselected | no |  |
| 274 | 66 | unselected | no |  |
| 275 | 66 | selected | no |  |
| 276 | 66 | unselected | no |  |
| 277 | 66 | unselected | no |  |
| 278 | 67 | selected | yes |  |
| 279 | 67 | selected | no |  |
| 280 | 67 | selected | yes |  |
| 281 | 67 | unselected | unknown |  |
| 282 | 67 | unselected | no |  |
| 283 | 67 | unselected | no |  |
| 284 | 68 | unselected | no |  |
| 285 | 68 | unselected | unknown |  |
| 286 | 68 | unselected | unknown |  |
| 287 | 68 | unselected | no |  |
| 288 | 68 | unselected | unknown |  |
| 289 | 68 | unselected | no |  |
| 290 | 68 | unselected | no |  |
| 291 | 69 | unselected | no |  |
| 292 | 69 | selected | no |  |
| 293 | 69 | unselected | no |  |
| 294 | 69 | unselected | no |  |
| 295 | 70 | selected | no |  |
| 296 | 70 | selected | yes |  |
| 297 | 70 | unselected | no |  |
| 298 | 70 | unselected | no |  |
| 299 | 74 | unselected | no |  |
| 300 | 75 | unselected | unknown |  |
| 301 | 76 | selected | no |  |
| 302 | 77 | selected | no |  |
| 303 | 78 | unselected | no |  |
| 304 | 78 | unselected | no |  |
| 305 | 81 | unselected | no |  |
| 306 | 82 | unselected | no |  |
| 307 | 87 | selected | yes |  |
| 308 | 89 | unselected | no |  |

**Supplementary Table 2:** Modified Manchester Scoring System

| **Cancer** | **Age at diagnosis** | **Score** |
| --- | --- | --- |
| Female Breast Cancer | <30 | 11 |
| Female Breast Cancer | 30-39 | 8 |
| Female Breast Cancer | 40-49 | 6 |
| Female Breast Cancer | 50-59 | 4 |
| Female Breast Cancer | >59 | 2 |
| Male Breast Cancer | <60 | 13 |
| Male Breast Cancer | >59 | 10 |
| Ovarian Cancer | <60 | 13 |
| Ovarian Cancer | >59 | 10 |
| Pancreatic Cancer |  | 1 |
| Prostate Cancer | <60 | 2 |
| Prostate Cancer | >59 | 1 |

The Manchester score is an empirical scoring system which estimates the chance of identifying a mutation in *BRCA1* or *BRCA2* . A score is assigned depending on the age and type of cancer in an individual. To calculate the Manchester score every individual in a direct lineage in a family is given a score from the above table. These scores are summed to give the total Manchester score. For further information please see [www.icr.ac.uk/protocols](http://www.icr.ac.uk/protocols).

**Breast Cancer Susceptibility Collaboration BCSC (UK)**

The following individuals are part of Breast Cancer Susceptibility Collaboration UK (BCSC) and coordinate recruitment of the families and sample collection. The centre coordinators are in bold.

A. Ardern-Jones, **J. Adlard**, G. Attard, K. Bailey, E. Bancroft, C. Bardsley, D. Barton, **J. Barwell**, L. Baxter, R. Belk, **J. Berg**, T. Bishop, L. Boyes, N. Bradshaw, **A. Brady**, S. Brant, **C. Brewer**, **G. Brice**, G. Bromilow, C. Brooks, A. Bruce, B. Bulman, L. Burgess, J. Campbell, B. Castle, R. Cetnarskyj, **C. Chapman**, O. Claber, N. Coates, T. Cole, A. Collins, **J. Cook**, S. Coulson, G. Crawford, D. Cruger, C. Cummings, L. D’Mello, **R. Davidson**, L. Day, L. de Silva, B. Dell, C. Dolling, **A. Donaldson**, A. Donaldson, H. Dorkins, **F. Douglas**, S. Downing, S. Drummond, J. Dunlop, S. Durrell, **D. Eccles**, C. Eddy, M. Edwards, E. Edwards, J. Edwardson, R. Eeles, I. Ellis, F. Elmslie, G. Evans, B. Gibbens, C. Gardiner, C. Giblin, S. Gibson, S. Goff, S. Goodman, D. Goudie, **L. Greenhalgh**, J. Greer, H. Gregory, D. Halliday, R. Hardy, C. Hartigan, T. Heaton, **A. Henderson,** C. Higgins, S. Hodgson, T. Holt, T. Homfray, D. Horrigan, C. Houghton, R.S. Houlston, L. Hughes, V. Hunt, L. Irvine, **L. Izatt**, L. Jackson, C. Jacobs, S. James, M. James, L. Jeffers, I. Jobson, W. Jones, M.J. Kennedy, S. Kenwrick, C. Kightley, C. Kirk, L. Kirk, E. Kivuva, **A. Kumar**, **F. Lalloo**, N. Lambord, C. Langman, P. Leonard, S. Levene, S. Locker, P. Logan, M. Longmuir, A. Lucassen, V. Lyus, A. Magee, A. Male, S. Mansour, D. McBride, E. McCann, V. McConnell, M. McEntagart, K. McDermot, C. McKeown, L. McLeish, D. McLeod, L. Mercer, C. Mercer, **Z. Miedzybrodzka**, J. Miller, A. Mitra, **P. J. Morrison**, V. Murday, A. Murray, J. Myring, **J. Paterson**, P. Pearson, G. Pichert, K. Platt, **M. Porteous**, C. Pottinger, S. Price, L. Protheroe, S. Pugh, O. Quarrell, C. Riddick, L. Robertson, A. Robinson, V. Roffey-Johnson, **M. Rogers**, S. Rose, S. Rowe, A. Schofield, N. Rahman, G. Scott, J. Scott, A. Searle, **S. Shanley**, S. Sharif, J. Shaw, J. Shea-Simonds, L. Side, J. Sillibourne, K. Simon, S. Simpson, S. Slater, K. Smith, L. Snadden, J. Soloway, Y. Stait, B. Stayner, M. Steel, C. Steel, H. Stewart, D. Stirling, M. Thomas, S. Thomas, S. Tomkins, H. Turner, E. Tyler, E. Wakeling, F. Waldrup, **L. Walker**, C. Watt, S. Watts, A. Webber, C. Whyte, J. Wiggins, E. Williams, L. Winchester.

**Triple Negative Trial Management Group (TNT TMG)**

The following individuals comprise the TNT Trial Management Group:

Andrew Tutt (Chair); Judith Bliss; Paul Ellis; Mark Harries; Alan Ashworth; Jorge Reis-Filho; Robert Leonard; David Miles; Sarah Pinder; Ian Smith; Cheryl Gillett; Peter Barrett-Lee; Philip Debruyne; Catherine Harper-Wynne; Andrew Wardley; Helena Earl; Susan O’Reilly; Rebecca Roylance; Alison Jones; Sarah Kernaghan; Lucy Kilburn; Lisa Rogers; Ashley Shaw; Max Parmar & Rose Thompson.

Additional members of the Biological Sub-Committee:

Ian Ellis; Nazneen Rahman & Peter Parker
